# The necessity of routine postoperative laboratory tests after total hip arthroplasty for hip fracture in a semi-urgent clinical setting

**DOI:** 10.1186/s10195-020-00559-3

**Published:** 2020-11-10

**Authors:** Xiang-Dong Wu, Jia-Cheng Liu, Yu-Jian Li, Jia-Wei Wang, Gui-Xing Qiu, Wei Huang

**Affiliations:** 1 Department of Orthopaedic Surgery, The First Affiliated Hospital of Chongqing Medical University, No. 1, Youyi Road, Yuanjiagang, Yuzhong District, Chongqing, 400016 China; 2 Department of Orthopaedic Surgery, Peking Union Medical College Hospital, Chinese Academy of Medical Sciences & Peking Union Medical College, Beijing, 100730 China

**Keywords:** Total hip arthroplasty, Hip fracture, Laboratory test, Anemia, Hypoalbuminemia

## Abstract

**Background:**

Recent studies suggest that routine postoperative laboratory tests are not necessary after primary elective total hip arthroplasty (THA). This study aims to evaluate the utility of routine postoperative laboratory tests in patients undergoing THA for hip fracture in a semi-urgent clinical setting.

**Materials and methods:**

This retrospective study included 213 consecutive patients who underwent primary unilateral THA for hip fractures. Patient demographics, clinical information, and laboratory tests were obtained from the electronic medical record system. Multivariate logistic regression analysis was performed to identify risk factors associated with abnormal laboratory test-related interventions.

**Results:**

A total of 207 patients (97.18%) had abnormal postoperative laboratory results, which were mainly due to anemia (190/213, 89.20%) and hypoalbuminemia (154/213, 72.30%). Overall, 54 patients (25.35%) underwent a clinical intervention, 18 patients received blood transfusion, and 42 patients received albumin supplementation. Factors associated with blood transfusion were long operative time and low preoperative hemoglobin levels. Factors associated with albumin supplementation were long operative time and low preoperative albumin levels. Of the 33 patients with abnormal postoperative creatinine levels, 7 patients underwent a clinical intervention. For electrolyte abnormalities, sodium supplementation was not given for hyponatremia, three patients received potassium supplementation, and one patient received calcium supplementation.

**Conclusions:**

This study demonstrated a high incidence of abnormal postoperative laboratory tests and a significant clinical intervention rate in patients who underwent THA for hip fracture in a semi-urgent clinical setting, which indicates that routine laboratory tests after THA for hip fracture are still necessary for patients with certain risk factors.

**Level of Evidence:**

Level III.

*Trial registration* Clinical trial registry number ChiCTR1900020690.

## Introduction

Along with the rapid aging of the global population, hip fractures continue to be a global concern. Hip fractures affect more than 2 million people annually, and this number is modestly projected to increase to 6.26 million worldwide by 2050 [1–3]. The appropriate timing of hip fracture surgery has long been debated and studied extensively, and the current guidelines indicate that surgery for hip fracture should be performed within 48 h after injury for favorable outcomes [4–10]. However, surgical procedures often need to be delayed in clinical practice. Notably, total hip arthroplasty (THA) for hip fracture continues to grow as a treatment choice, as evidenced by trends in data from the USA, and a recent study indicated that the time to surgery does not affect mortality, revision for any reason, or the implant failure rate in patients undergoing THA [11, 12]. Compared with patients who underwent primary elective non-hip fracture THA, patients who underwent THA for hip fracture represent a high-risk group with concomitant medical comorbidities [13–18]. As a regional tertiary referral center, plenty of frail elderly patients with hip fracture and multiple comorbidities are transferred to our department. The majority of these patients are medically complex and surgery is usually postponed to allow for detailed medical evaluations to ensure that patients’ conditions are stable, and for preoperative correction (e.g., anemia, hypoalbuminemia) and medical optimization (e.g., pulmonary reserve, nutrition). Therefore, THA for hip fracture is treated as a semi-elective surgery in our routine clinical practice.

Several studies have recently evaluated the necessity of routine postoperative laboratory tests after primary elective total joint arthroplasty (TJA) [19–27]. These studies suggested that routine postoperative laboratory tests are not necessary for modern-day primary unilateral TJA, and only patients with specific risk factors should undergo postoperative laboratory tests [19–27]. The objective of this retrospective study is to assess the utility of routine postoperative laboratory tests after THA for hip fracture, after preoperative optimization in a semi-urgent clinical setting. We hypothesize that routine postoperative laboratory tests are unnecessary after THA in relatively healthy patients with hip fracture but may still be necessary for hip fracture patients with severe medical comorbidities.

## Materials and methods

Ethical approval for this study was obtained from our institutional ethical committee. This study complied with the Declaration of Helsinki and was approved by our institutional review board. The study protocol was registered in the International Clinical Trials Registry Platform (Registry Number: ChiCTR1900020690) [28].

### Study design and population

For this retrospective analysis, we included consecutively treated patients who underwent primary unilateral THA for hip fractures between January 2016 and November 2018 at a single institution. Adult patients who suffered from femoral neck fracture or intertrochanteric fracture and underwent primary unilateral THA were screened for potential eligibility. The exclusion criteria were as follows: (1) having undergone hemiarthroplasty, (2) having undergone THA for failed fixation of hip fractures, (3) having undergone THA for pathologic hip fractures or pathologic fractures around the hip, (4) having a history of a bleeding disorder, and (5) operative times over 240 min or less than 20 min (to limit the influence of extreme outliers) [28].

### Data collection

Demographic data and data related to the surgery and laboratory tests were retrieved from the electronic medical record system and entered in a standardized form (Excel 2019, Microsoft Corporation, Redmond, WA, USA). The following information was collected for each eligible patient: age, sex, body mass index (BMI), preoperative comorbidities, American Society of Anesthesiologists (ASA) physical status classification, operative time (defined as the time from the skin incision to wound closure), intraoperative blood loss, use of tranexamic acid (TXA), length of hospital stay, preoperative and postoperative laboratory test results [complete blood count (CBC) and comprehensive metabolic panel (CMP)], and any medical intervention directly related to abnormal laboratory test results.

### Study outcomes

The predefined outcomes included any medical interventions administered in response to abnormal postoperative laboratory test results, such as anemia prompting blood transfusion, hypoalbuminemia prompting albumin supplementation, and electrolyte disturbances requiring correction, hospitalist/specialist consultations, the initiation or discontinuation of medication, or additional scheduled laboratory tests.

### Perioperative management

In our institution, hip fracture patients who are admitted for THA routinely undergo extensive medical evaluations to determine their clinical status and ensure that their conditions are sufficiently stable to tolerate surgery. Usually, some patients with hip fractures have one or more chronic comorbidities or physiologic decompensation, and some of them require medical correction prior to surgery. In our clinical practice, patients who presented with preoperative anemia are usually treated with recombinant human erythropoietin (rHuEPO) and intravenous iron, unless they meet the criterion for blood transfusion [hemoglobin (Hb) level of < 70 g/L or symptomatic anemia with a Hb level > 70 g/L]. Based on our enhanced recovery after surgery (ERAS) protocol, a doctor from the Department of Nutrition is responsible for assessing patients’ nutritional status and preparing small-peptide and whole-protein formulas, which are orally administered perioperatively to improve patients’ nutritional status and correct hypoalbuminemia. Albumin supplementation is also not routinely given to patients with preoperative hypoalbuminemia (30 ≤ albumin concentration < 35 g/L), unless they meet the criterion for albumin supplementation (albumin concentration < 30 g/L). For preoperative electrolyte disturbances, patients with mild or moderate abnormalities usually receive orally administered mediation (food or tablet), and only patients with severe depletion receive electrolyte supplementation (Additional file 1: Material 1) [28–31]. If patients received a blood transfusion or albumin supplementation before surgery, a retest after transfusion was mandatory to assess the response and guide whether further transfusions are required, and the preoperative retest results would be used for data analysis.

Of note, in the operating room and postanesthesia care unit (PACU), blood gas analysis was performed on arterial blood samples, the results of which were also used to guide fluid and electrolyte supplement management [28].

### Statistical analysis

For quantitative data, the mean ± standard deviation (SD) or median (interquartile range) was calculated, and comparisons between groups were performed using the *t*-test or Wilcoxon rank-sum test, as appropriate. For qualitative data, the frequency or percentage was calculated, and group comparisons were performed using the chi-square test or Fisher’s exact test. Multivariate regression models were generated to identify variables that had the strongest associations with abnormal postoperative laboratory tests necessitating a medical intervention, and only candidate variables with a *P* value of not more than 0.1 in the univariate analysis were included in a multivariate model. The results are presented using the odds ratio (OR) with 95% confidence interval (CI). *P* < 0.05 was considered statistically significant (two-tailed). All statistical analyses were performed using SPSS, version 21.0 software (SPSS Inc., Chicago, IL, USA).

## Results

### Patient characteristics

Of the 690 potentially eligible patients, a total of 213 hip fracture patients were identified, including 155 (72.77%) female and 58 (27.23%) male patients. Of the included patients, 158 (74.18%) patients received uncemented THA and 55 (25.82%) patients received hybrid THA. The patient demographics and perioperative data are presented in **Table ****1**. The incidences of preoperative anemia and hypoalbuminemia were 41.78% (89/213) and 10.33% (22/213), respectively, in the preoperative period (Table 2). However, after surgery, 207 patients (97.18%) had abnormal postoperative laboratory results, which were mainly due to anemia (190/213, 89.20%) and hypoalbuminemia (154/213, 72.30%). Overall, 54 patients (25.35%) with abnormal postoperative laboratory test results necessitated clinical intervention (Tables 3 and 4).

**Table 1:** Baseline characteristics of the study cohorts

Variables	Total	Normal postoperative labs	Abnormal postoperative labs	*P* value
N (%)	213 (100%)	6 (11.6%)	207 (88.4%)	
Preoperative variables				
Age (years)	72.7 ± 10.7	57.5 ± 9.3	73.2 ± 10.4	< 0.001
Gender	155F:58 M	3F:3 M	152F:55 M	0.348
BMI (kg/m^2^)	22.4 ± 3.6	23.8 ± 2.1	22.4 ± 3.6	0.377
ASA score	2.8 ± 0.6	2.3 ± 0.5	2.8 ± 0.6	0.057
Diabetes	39	0	39	0.595
Anemia	89	0	89	0.042
Hemoglobin level (g/L)	119.6 ± 16.0	139.0 ± 9.7	119.0 ± 15.9	0.002
Hypoalbuminemia	22	0	22	0.871
Albumin Level (g/L)	39.4 ± 4.5	43.3 ± 1.9	39.3 ± 4.5	0.029
Abnormal creatinine	35	0	35	0.592
Creatinine level (μmol/L)	77.0 ± 52.4	73.7 ± 11.5	77.1 ± 53.1	0.875
Abnormal sodium	16	0	16	> 0.999
Sodium level (mmol/L)	140.9 ± 3.0	143.3 ± 1.8	140.9 ± 3.0	0.043
Abnormal potassium	19	0	19	0.959
Potassium level (mmol/L)	4.0 ± 0.4	4.1 ± 0.3	4.0 ± 0.5	0.783
Abnormal calcium	27	1	26	0.561
Calcium level (mmol/L)	2.25 ± 0.12	2.36 ± 0.10	2.25 ± 0.12	0.031
Preoperative LOS (days)	6.1 ± 3.7	4.2 ± 1.0	6.2 ± 3.7	0.189
Intraoperative variables				
TXA use	192	6	186	0.787
Estimated blood loss (mL)	117.0 ± 93.5	75.0 ± 27.4	118.2 ± 94.5	0.266
Operation time (min)	69.0 ± 30.6	48.2 ± 16.6	69.6 ± 30.8	0.092
Postoperative variables				
Drop in hemoglobin (g/L)	17.6 ± 11.0	8.5 ± 7.6	17.9 ± 11.0	0.040
Drop in albumin (g/L)	7.1 ± 4.1	4.7 ± 2.7	7.1 ± 4.1	0.147
Drop in creatine (μmol/L)	1.8 ± 20.6	6.7 ± 5.6	1.6 ± 20.8	0.553
Drop in sodium (mmol/L)	1.1 ± 3.1	2.3 ± 1.9	1.1 ± 3.2	0.326
Drop in potassium (mmol/L)	− 0.34 ± 0.52	− 0.32 ± 0.47	− 0.34 ± 0.53	0.930
Drop in calcium (mmol/L)	0.16 ± 0.14	0.17 ± 0.11	0.18 ± 0.14	0.876
Postoperative LOS (days)	8.46 ± 3.5	6.0 ± 0.9	8.5 ± 3.6	< 0.001

*N* number, *F* female, *M* male, *BMI* body mass index, *ASA* American Society of Anesthesiologists, *LOS* length of stay, *TXA* tranexamic acid

**Table 2:** Abnormal preoperative laboratory tests in different patient groups

Preoperative item	Total (*n* = 213) (%)	Normal postoperative labs group (*n* = 6)	Abnormal postoperative lab group (*n* = 207)	Abnormal preoperative lab without intervention group (*n* = 153)	Abnormal postoperative lab requiring intervention group (*n* = 54)
Diabetes	39 (18.31%)	0	39	25	14
Anemia	89 (41.78%)	0	89	56	33
Hypoalbuminemia	22 (10.33%)	0	22	7	15
Abnormal creatinine	35 (16.43%)	0	35	18	17
Abnormal sodium	16 (7.51%)	0	16	10	6
Abnormal potassium	19 (8.92%)	0	19	12	7
Abnormal calcium	27 (12.68%)	1	26	11	15

**Table 3:** Comparison of variables between patients with abnormal postoperative laboratory tests and with or without intervention

Variables	Abnormal postoperative lab without intervention	Abnormal postoperative lab with intervention	*P* value
*N* (%)	153	54	
Preoperative variables			
Age (years)	72.0 ± 10.4	76.6 ± 9.9	0.005
Gender	110F:43 M	42F:12 M	0.400
BMI (kg/m^2^)	22.7 ± 3.7	21.4 ± 3.2	0.039
ASA score	2.8 ± 0.6	3.0 ± 0.6	0.025
Diabetes	25	14	0.121
Anemia	56	33	0.002
Hemoglobin level (g/L)	121.8 ± 14.2	111.1 ± 17.6	< 0.001
Hypoalbuminemia	7	15	< 0.001
Albumin level (g/L)	40.1 ± 4.2	37.0 ± 4.5	< 0.001
Abnormal creatinine	18	17	0.001
Creatinine level (μmol/L)	72.3 ± 42.2	90.8 ± 74.7	0.027
Abnormal sodium	10	6	0.279
Sodium level (mmol/L)	141.1 ± 2.9	140.2 ± 3.1	0.045
Abnormal potassium	12	7	0.263
Potassium level (mmol/L)	4.0 ± 0.4	4.0 ± 0.5	0.987
Abnormal calcium	11	15	< 0.001
Calcium level (mmol/L)	2.26 ± 0.10	2.22 ± 0.16	0.113
Preoperative LOS (days)	5.9 ± 3.8	7.0 ± 3.5	0.061
Intraoperative variables			
TXA use	138	48	0.784
Estimated blood loss (mL)	102.6 ± 61.0	163.9 ± 144.8	< 0.001
Operation time (min)	64.8 ± 24.1	83.2 ± 41.9	0.003
Postoperative variables			
Drop in hemoglobin (g/L)	17.5 ± 9.8	18.8 ± 13.9	0.521
Drop in albumin (g/L)	6.9 ± 4.0	7.9 ± 4.5	0.099
Drop in creatine (μmol/L)	1.93 ± 10.71	0.67 ± 36.81	0.804
Drop in sodium (mmol/L)	1.1 ± 3.0	0.8 ± 3.5	0.544
Drop in potassium (mmol/L)	− 0.34 ± 0.50	− 0.33 ± 0.59	0.945
Drop in calcium (mmol/L)	0.15 ± 0.12	0.19 ± 0.18	0.138
Postoperative LOS (days)	8.1 ± 3.4	9.7 ± 3.9	0.004

*N* number, *F* female, *M* male, *BMI* body mass index, *ASA* American Society of Anesthesiologists, *LOS* length of stay, *TXA* tranexamic acid

**Table 4:** Abnormal postoperative laboratory tests and intervention directly related to abnormal laboratory tests in the study cohorts

Postoperative lab test (*n* = 213)	Abnormal postoperative lab (*n* = 207)	Abnormal postoperative lab without intervention (*n* = 153)	Abnormal postoperative lab requiring intervention (*n* = 54)	Received clinical intervention
Anemia	190	139	51	18
Hypoalbuminemia	154	104	50	42
Abnormal creatinine	33	20	13	7
Abnormal sodium	23	15	8	0
Abnormal potassium	11	5	6	3
Abnormal calcium	117	75	42	1

### Hb

Of the 190 patients with postoperative anemia, 18 patients received blood transfusions, and they were compared with those who did not require blood transfusion. The univariate analysis detected significant differences between the two groups of patients in the ASA score, operative time, and preoperative Hb level. Multivariate logistic regression analysis showed that long operative time (OR 1.018, 95% CI 1.003–1.033, *P* = 0.015) and low preoperative Hb levels (OR 0.950, 95% CI 0.913–0.989, *P* = 0.012) were independent risk factors for postoperative blood transfusion (Additional file 2: Material S2).

### Albumin

For abnormal postoperative albumin levels, univariate analysis showed that age, BMI, ASA score, operative time, and preoperative albumin levels were associated with postoperative albumin supplementation. Multivariate logistic regression revealed that long operative time (OR 1.013, 95% CI 1.001–1.024, *P* = 0.035) and low preoperative albumin level (OR 0.757, 95% CI 0.673–0.852, *P* < 0.001) were independent risk factors for postoperative albumin supplementation.

### Creatinine

Although 33 (15.49%) patients had abnormal postoperative creatinine levels, most of whom were asymptomatic with no adverse events, only 1 patient undergoing regular hemodialysis treatment required additional dialysis. One patient was administered a fluid bolus, two patients were administered NiaoDuQing granules (a traditional Chinese medicine) after consultation, and three patients underwent clinical observation only after consultation.

### Electrolyte disturbances

Hyponatremia was responsible for all Na abnormalities, but Na supplementation was not administered. Regarding K abnormalities, five patients had mild hypokalemia (3.0–3.5 mmol/L), three of whom received K supplementation, five patients had mild hyperkalemia (5.4–6.0 mmol/L), all of whom did not receive any treatment, and one patient had moderate hyperkalemia (6.0–6.4 mmol/L) and required an additional hemodialysis treatment as described previously. A total of 116 (54.46%) patients had postoperative hypocalcemia, but only 1 patient received postoperative calcium supplementation, one patient with mild hypercalcemia received no treatment. Multiple logistic regression analysis could not be performed due to the very low intervention rates for abnormal postoperative creatinine, Na, K, and Ca levels (Additional file 3: Material S3).

## Discussion

### Main findings

To the best of the authors’ knowledge, this study represents the first attempt to evaluate the utility of routine postoperative laboratory tests after THA for hip fracture in a semi-urgent clinical setting. In this study, a high incidence of abnormal postoperative laboratory test results and a substantial intervention rate after THA for hip fracture patients were noted in the included patients, and risk factors for blood transfusion and albumin supplementation were identified. The findings suggest that routine postoperative laboratory tests for certain patients with risk factors are justifiable.

### Comparison with previous studies

Recently, several studies on this topic have been published; however, the results of this study conflict with those of previous similar studies [24, 26, 27]. In contrast, previous research mainly focused on primary elective THA patients, which are significantly different from hip fracture patients who undergo semi-elective THA. In general, patients undergoing THA for acute hip fractures are at a substantially higher risk of medical comorbidities and complications than patients undergoing primary elective THA [18, 31–34]. Compared with non-hip fracture patients, hip fracture patients represent a high-risk population with older age, female predominance, lower BMI, poorer nutritional status, more concomitant medical comorbidities, and more preoperative abnormalities (Table 5). These differences accounted for most of the differences in the outcomes between the two subpopulations.

**Table 5:** Comparison of patient characteristics with previous similar studies

Study	Country	No. of patients	Clinical setting	Female (% of total)	Age (years)	BMI (kg/m^2^)	Preoperative anemia	Transfusion rate
Greco et al.^26^/2019	USA	401	Elective THA for non-hip fracture	60.35%	71.0 ± 7.6	30.0 ± 6.5	–	2.49% (10/401)
Halawi et al.^24^/2019	USA	351	Elective THA for non-hip fracture	47.01%	57.8 ± 11.2	30.5 ± 6.0	17.38% (61/351)	2.30% (8/351)
Wu et al.^27^/2020	China	395	Elective THA for non-hip fracture	51.39%	58.2 ± 13.1	24.0 ± 3.6	26.3% (104/395)	1.77% (7/395)
Present study	China	213	Semi-elective THA for hip fracture	72.77%	72.7 ± 10.7	22.4 ± 3.6	41.78% (89/213)	8.45% (18/213)

*BMI* body mass index, *THA* total hip arthroplasty

Although patient characteristics may explain the high incidence rates of abnormal postoperative laboratory tests and clinical intervention, the perioperative clinical pathway and physiological processes associated with hip fracture (e.g., the acute inflammatory stress, catabolic states) may also account for some of the high incidences [18, 35, 36]. The clinical pathway for THA differs between non-hip patients and hip fracture patients and can vary among institutions, which may lead to diverse results.

### Implications for clinical practice

Although clinical guidelines recommend surgical treatment within 48 h after hip fracture, in clinical practice, hip fracture surgery is often postponed for potential benefits from preoperative optimization, but also maybe due to the limited capacity of operating rooms or personnel [37]. This delay in processes may partially account for some of the perioperative morbidity and abnormal postoperative laboratory tests; therefore, these may represent modifiable risk factors. Even though hip fracture patients tend to have more preoperative abnormalities and a higher burden of comorbidities than non-hip patients, preoperative medical stabilization and optimization are often not associated with relevant interventions, as most of the abnormal preoperative laboratory test results do not meet the criteria for clinical interventions. As hip fracture surgery should be performed early to minimize exposure to the intrinsic harmful factors of fracture, relevant treatment interventions should be implemented promptly to improve the clinical state of patients. Additional studies are still warranted to balance the benefits and risks of early surgery for mobilization and delayed surgery for preoperative optimization.

The operative time was found to be an independent predictor for blood transfusion and albumin supplementation, and it is a readily available measure that can aid in risk stratification and decision-making regarding postoperative laboratory test results [38–40]. In effect, although THA involves many standardized surgical procedures and cannot be unnecessarily shortened, many avoidable factors can introduce intraoperative delay, which includes but not limited to the surgeons’ experience (e.g., surgical resident, senior surgeon, and consultant), preoperative planning, timing of the surgery (e.g., nights, weekends, or holidays), and compatibility of the multidisciplinary team [41]. Therefore, surgeon training and experience, preoperative planning, and procedure efficiency should be optimized to minimize the operative time.

THA is characterized by significant blood loss, and blood transfusions have always been inevitable, especially among hip fracture patients. The transfusion rate was once as high as 30–70%, but has fallen precipitously since the 2010s; it is as low as 9% for THA [42–46]. Blood conservation strategies, including new surgical techniques, Hb optimization, induced hypotension, and in particular, application of TXA, have fundamentally changed perioperative blood management [18, 23–25, 28, 47]. Therefore, the incidences of postoperative anemia and related transfusion were much lower than they were before, and the values of postoperative CBC became reasonably limited. Our study found that the transfusion rate was 8.45%, and the risk factors for transfusion were long operative time and low preoperative Hb levels.

It is noticeable, however, that hypoalbuminemia presents another severe challenge of THA for hip fracture patients. Hypoalbuminemia is not synonymous with malnutrition or undernutrition, but is highly related to it. Hypoalbuminemia has been associated with increased perioperative morbidity and mortality, and in general, cardiac, as well as orthopedic surgery [48–52]. In our study, 72.30% of the patients developed hypoalbuminemia after THA, and 19.72% of the patients met the criteria and received albumin supplementation. We detected that long operative time and low preoperative albumin levels were independent risk factors for postoperative albumin supplementation. As diet plays an important role in nutrition status, multimodel nutritional management, especially perioperative oral nutritional supplementation, would improve the level of serum albumin, and albumin infusion remains essential and beneficial for patients with malnutrition or undernutrition [53, 54].

Previous research has fully expounded that chronic kidney disease (CKD) is a significant risk factor for abnormal postoperative creatinine levels and acute kidney injury (AKI) [22, 55, 56]. For patients with definitive preoperative diagnosis of CKD, preoperative assessment of renal function remains essential, and nephrotoxic agents should be reduced or avoided to prevent induced AKI [22]. In the present study, most abnormal patients experienced flare-ups of the creatinine level without clinical symptoms or related complications.

Mild forms of electrolyte disturbance may not cause any symptoms and often go undetected until they are discovered during a routine basic metabolic panel (BMP) test. Nevertheless, clinical symptoms usually start to appear once the severity of a particular disorder has increased. Currently, information on the prognosis of patients with hyponatremia who have undergone THA is limited, and the vast majority of patients neither have any symptoms nor receive any interventions. Previous studies have indicated that the baseline Na levels, age, and not using TXA are significant outcome predictors [22–24]. The incidences of abnormal Na level (10.80% versus 41.3%) and intervention rate (0% versus 14.5%) were significantly lower in our study than in a previous study, which may mainly be attributed to differences in postoperative intravenous infusion and the common use of normal saline as an infusion solution [22].

For abnormal serum K levels, symptoms of hypokalemia or hyperkalemia, such as cardiac arrhythmias, have been fully described. Clinical interventions related to hypokalemia or hyperkalemia tend to be more aggressive, and no patient presented with atypical symptoms or reported discomfort. Previous studies have indicated that abnormal baseline K values, anemia, female sex, diabetes, and CKD are risk factors for abnormal postoperative K values [22–24, 26]. The incidence of abnormal K levels (5.16% versus 25.2%) and intervention rate (1.41% versus 32.6%) were also significantly lower in our study, which may mainly be attributed to differences in intraoperative fluid management and the correction of electrolyte disturbance [22, 28].

Regarding abnormal serum calcium levels, postoperative hypocalcemia might trigger cardiac arrhythmia events and induce blood coagulation dysfunction. Although hypocalcemia frequently occurs after THA (54.93%), clinical symptoms associated with hypocalcemia are extremely rare [57]. Routinely, no intervention would be applied for hypocalcemia, but there is a limited understanding of hypocalcemia in patients after THA, thus warranting additional studies [58].

### Strengths and limitations

A major strength of this study is that it represents the first investigation of the necessity of routine postoperative laboratory tests after THA for hip fracture in a semi-urgent clinical setting. This study also had several limitations that affected the interpretation of the results, beginning with the retrospective nature of the study design. The small number of patients included from a single tertiary academic center, particularly in a semi-urgent clinical setting, may limit the generalizability of these findings. Next, the clinical management of patients in response to postoperative laboratory abnormalities may vary among surgeons, and decisions to adopt interventions were made to some extent based on the clinical judgment of each surgeon. In addition, other data (e.g., comorbidities and related medications) and events (e.g., perioperative complications) were not collected from the electronic medical record system, thereby limiting additional analyses. Last, follow-up data, including consultations, complications, and mortality rates were not available.

## Conclusions

This study showed a high occurrence of abnormal postoperative laboratory tests and a significant clinical intervention rate, which provides evidence that the routine practice of obtaining postoperative laboratory tests after THA for hip fracture is still necessary for certain patients. Long operative time and low preoperative Hb levels increase the risk of blood transfusion, whereas long operative time and low preoperative albumin levels increase the risk of albumin supplementation. However, the majority of abnormalities are inconsequential.

## Grant disclosures

This study received no specific grant from any funding agency, commercial, or not-for-profit sectors.

## Supplementary information

**Additional file 1: Material S1.** Reference ranges for complete blood count and comprehensive metabolic panel and corresponding threshold values for clinical intervention.

**Additional file 2: Material S2.** The necessity of routine postoperative laboratory tests for enhanced recovery after surgery for primary hip and knee arthroplasty.

**Additional file 3: Material S3.** IRB approval.

## Data Availability

The datasets used and analyzed during the study are available from the corresponding author on reasonable request.

## Abbreviations

AKI: Acute kidney injury
ASA: American Society of Anesthesiologists Physical Status Classification
BMI: Body mass index
BPM: Basic metabolic panel
CI: Confidence interval
CKD: Chronic kidney disease
CMP: Comprehensive metabolic panel
ERAS: Enhanced recovery after surgery
F: Female
Hb: Hemoglobin
LOS: Length of stay
M: Male
N: Number
OR: Odds ratio
PACU: Postanesthesia care unit
rHuEPO: Recombinant human erythropoietin
SD: Standard deviation
THA: Total hip arthroplasty
TJA: Total joint arthroplasty
TXA: Tranexamic acid
U.S.: United States
