# Supplementary material for: The necessity of routine postoperative laboratory tests after total hip arthroplasty for hip fracture in a semi-urgent clinical setting
Source: J Orthop Traumatol. 2020 Nov 10;21:19. doi: 10.1186/s10195-020-00559-3 (PMC7655881; doi:10.1186/s10195-020-00559-3)
Supplement: Supplementary file 1 — Additional file 1: Material S1. Reference ranges for complete blood count and comprehensive metabolic panel and corresponding threshold values for clinical intervention. [file 10195_2020_559_MOESM1_ESM.pdf]

Study Protocol Clinical Trial

Medicine®

OPEN

The necessity of routine postoperative laboratory tests in enhanced recovery after surgery for primary hip and knee arthroplasty

A retrospective cohort study protocol

Xiang-Dong Wu, MD*, Peng-Cheng Xiao, MD, Zheng-Lin Zhu, MD, Jia-Cheng Liu, MD, Yu-Jian Li, MD, Wei Huang, MD, PhD*

Abstract

Introduction: Over the last few decades, the concepts of minimally invasive surgery and enhanced recovery after surgery (ERAS) protocols have been introduced into the field of total joint arthroplasty (TJA), and tranexamic acid (TXA) has been widely used in TJA. Modern-day surgical techniques and perioperative care pathways of TJA have experienced unexpected improvements. Recently, the necessity of the practice of ordering routine postoperative laboratory tests for patients undergoing primary TJA has been challenged, especially in the context of implementation of ERAS protocols in TJA. These studies have consistently suggested that routine postoperative laboratory tests are not necessary in modern-day primary, unilateral total hip arthroplasty (THA) or total knee arthroplasty (TKA), and laboratory tests after surgery should only be obtained for patients with risk factors. However, it remains unclear whether routine postoperative laboratory tests after THA and TKA remains justified in the Chinese patient population. Therefore, we developed this study to address this issue.

Methods and analysis: This retrospective cohort study will include adult patients who underwent primary unilateral THA or TKA and received multimodal perioperative care pathways according to ERAS protocols. The following patient data will be collected from the electronic medical record system: patients' demographics, preoperative and postoperative laboratory values, operation time, intraoperative blood loss, TXA use, tourniquet use, postoperative length of stay, and any medical intervention directly related to abnormal laboratory values. The main study outcomes are the incidence of acute anemia requiring transfusion and incidence of hypoalbuminemia requiring albumin supplementation. The secondary outcomes are the rates of acute kidney injury, incidence of abnormal serum sodium level, incidence of abnormal serum potassium level, and incidence of abnormal serum calcium level. These clinical data will be analyzed to determine the incidence of abnormal postoperative laboratory values following primary unilateral THA and TKA; to clarify the frequency of any medical intervention directly related to abnormal postoperative laboratory values; and to identify risk factors that predispose patients to have abnormal postoperative laboratory results.

Study registration: Chinese Clinical Trial Registry (http://www.chictr.org.cn): ChiCTR1900020690.

Abbreviations: Alb = albumin, BMI = body mass index, Ca = calcium, Cre = creatinine, ERAS = enhanced recovery after surgery, Hb = hemoglobin, K = potassium, MIS = minimally invasive surgery, Na = sodium, PLT = platelet count, THA = total hip arthroplasty, TJA = total joint arthroplasty, TKA = total knee arthroplasty, TXA = tranexamic acid.

Keywords: enhanced recovery after surgery, postoperative laboratory tests, total hip arthroplasty, total knee arthroplasty, tranexamic acid

Downloaded from https://journals.lww.com/md-journal by BhDMf5ePHKav1zEoum1tQfN4a+kJLhEZgbsIHo4XMi0hCywCX1AWnYQp/IlQrHD3gDUFDIs0jTnMaD+4QF0p6AgX78a9dhS7qGhBMFKlg= on 05/04/2019

X-DW, P-CX, and Z-LZ are co-first authors.

This study has been approved by The First Affiliated Hospital of Chongqing Medical University Ethics Committee. The findings of this study will be disseminated at national and international conferences, and will also be published in peer-reviewed, scientific journals.

Ethical approval for the study was obtained through The First Affiliated Hospital of Chongqing Medical University.

The datasets used and/or analyzed during the present study are available from the corresponding author on reasonable request.

This study was supported by Special Fund for Local Scientific and Technological Development under the Guidance of the Central Government (Grant No. Z135050009017).

All the authors declare that they have no conflict of interest with any organization, and this research received no specific grant from any funding agency, commercial or not-for-profit sectors.

Department of Orthopaedic Surgery, The First Affiliated Hospital of Chongqing Medical University, Chongqing, China.

** Correspondence: Xiang-Dong Wu, Department of Orthopaedic Surgery, The First Affiliated Hospital of Chongqing Medical University, No. 1, Youyi Road, Yuanjiagang, Yuzhong District, Chongqing 400016, China (e-mail: xiangdong_wu@outlook.com), Wei Huang, Department of Orthopaedic Surgery, The First Affiliated Hospital of Chongqing Medical University, No. 1, Youyi Road, Yuanjiagang, Yuzhong District, Chongqing 400016, China (e-mail: drhuangwei68@gmail.com).*

Copyright © 2019 the Author(s). Published by Wolters Kluwer Health, Inc.
This is an open access article distributed under the Creative Commons Attribution License 4.0 (CCBY), which permits unrestricted use, distribution, and reproduction in any medium, provided the original work is properly cited.

Medicine (2019) 98:18(e15513)

Received: 8 April 2019 / Accepted: 12 April 2019

http://dx.doi.org/10.1097/MD.0000000000015513

1. Introduction

Total joint arthroplasty (TJA) has been shown to be one of the most successful surgical interventions in medicine, and total hip arthroplasty (THA) has been widely acclaimed as the "Operation of The Century."[1,2] However, THA and total knee arthroplasty (TKA) appear to be more successful in recent years. During the past decades, THA and TKA have traditionally been performed through larger incisions and associated with extensive injuries to deep tissue and underlying structures, extensive soft tissue dissections, massive perioperative blood loss, high transfusion rate, more severe acute postoperative pain, a high risk for complications, extended hospital stay, and delayed rehabilitation.[3–18]

Therefore, minimally invasive surgery (MIS) techniques for THA and TKA which aimed to achieve a smaller incision and less soft tissue disruption, were initially introduced to orthopedic surgeons in the 1990s.[4–6,19,20] The continuous development of MIS instrumentation and implants, as well as sophisticated digital navigation technologies, has led to the rapid advancement of less invasive surgical techniques.[21–23] A number of studies and systematic reviews have indicated that MIS techniques led to a significant decrease in surgical duration, blood loss, pain, hospital stay, and recovery time.[24–32]

Meanwhile, the concept of enhanced recovery after surgery (ERAS) was advocated in the 1990s and gradually introduced to the field of joint arthroplasty.[33–35] ERAS protocols for TJA include a continuous procedure-specific analysis of the various core components that influence the stress response and enhance recovery, including the choice of optimal anesthetic, MIS technique, pain management, and perioperative blood management, thromboembolic prophylaxis, and adjustment of care principles (drains, catheters, monitoring, etc).[33–38] There is abundant evidence that multidisciplinary perioperative management has shown efficacy in minimizing tissue trauma, improving blood conservation, reducing pain, reducing complications, shortening hospital stay, accelerating return of function, reducing costs, and improving patient comfort and satisfaction.[34–40] Of note, the use of tranexamic acid (TXA) in TJA, which extensively reduced perioperative blood loss and risk of transfusion, is another successful milestone for blood conservation, fundamentally changing blood management by making transfusion an infrequent event.[41–46]

The concepts of MIS and ERAS and the routine use of TXA in TJA were not yet introduced into the field of TJA until the previous two decades. Modern-day surgical techniques and perioperative care pathways for TJA have experienced unexpected improvements. Recently, several studies have questioned the necessity of postoperative laboratory tests (e.g., complete blood count; comprehensive metabolic panel), which are routinely ordered for patients after TJA.[47–53] These retrospective studies have consistently suggested that routine postoperative laboratory tests are not necessary in modern-day primary unilateral THA or TKA. Instead, only patients with risk factors should undergo laboratory tests after surgery.[48–52]

Considering that the Chinese patients population who received THA and TKA tend to be older, with a lower mean body mass index (BMI) and higher incidence of anemia and malnutrition, the generalizability of these studies is limited, and it is unclear whether routine postoperative laboratory tests after THA and TKA remain justified in China. Therefore, re-evaluating the utility of routine postoperative laboratory tests after primary unilateral THA and TKA in a Chinese population is warranted. The overarching aims of the study are to estimate the necessity of routine postoperative laboratory tests in ERAS for primary unilateral THA and TKA; to determine the incidence of abnormal postoperative laboratory values following primary unilateral THA and TKA; to clarify the frequency of any medical intervention directly related to abnormal laboratory values; and to identify risk factors that predispose patients to have abnormal postoperative laboratory results.

2. Methods and analysis

This study will be performed and reported in accordance with the STrengthening the Reporting of OBservational studies in Epidemiology checklist.[54]

2.1. Study design

This is a 3-year retrospective cohort study, which will be conducted in a tertiary referral center—The First Affiliated Hospital of Chongqing Medical University. It will run from January 2016 (the beginning of the implementation of the ERAS protocol in our department) to November 2018. The study population will be separated into 2 sections: patients who underwent primary unilateral THA and patients who underwent primary unilateral TKA. This separation is based on our institutional experience: compared with patients who undergo THA, patients who undergo TKA tended to be healthier with less pre-existing comorbidities, and more active before surgery. In addition, both intraoperative blood loss and perioperative blood loss in TKA are lower than in THA.[55] Theoretically, the percentage of patients with abnormal postoperative laboratory values after primary unilateral TKA should be fairly limited. We will further compare the incidence of abnormal postoperative laboratory values and frequency of any medical intervention directly related to abnormal laboratory values between THA and TKA to corroborate this inference. The study protocol is registered at http://www.chictr.org.cn (Chinese Clinical Trial Registry ID: ChiCTR1900020690).

2.2. Study population

2.2.1. Inclusion criteria. Adult patients who underwent primary unilateral THA or TKA and received multi-modal perioperative care pathways according to the ERAS protocol will be screened for potential eligibility.

2.2.2. Exclusion criteria. A subject who meets any of the following criteria will be excluded from participation in this study:

(1) simultaneous bilateral THA or TKA;
(2) hip resurfacing arthroplasty or hemiarthroplasty;
(3) partial knee arthroplasty (including unicompartmental knee arthroplasty, bicompartmental knee arthroplasty, and patellofemoral arthroplasty);
(4) revision of THA or TKA;
(5) undergone primary unilateral THA or TKA after bone tumor resection of the hip or knee;
(6) with a previous diagnosis of an inherited bleeding disorder;
(7) in order to limit the influence of extreme outliers, cases with recorded operative times greater than 240 minutes or less than 20 minutes would also be excluded.[56,57]

Table 1

Reference ranges for complete blood count and theoretically threshold values for clinical intervention.

	Reference range		
Component	Male	Female	Theoretically threshold values for clinical intervention
Hemoglobin, g/L	130–175	115–150	Hemoglobin level of <70 g/L or symptomatic anemia with a hemoglobin level >70 g/L
Hematocrit %	40–50	35–45	–
Platelet count 10^9/L	85–303	101–320	Platelet count <30*10^9/L poor platelet function

2.2.3. Perioperative management. During the study period, 1.5 g TXA was routinely administered intravascularly (IV) 30 minutes before incision, another dose of 1g topical (intra-articular) TXA was injected into the wound before skin closure, but the dosage of postoperative IV TXA varied. For the purpose of our analysis, we would include all patients who received TXA regardless of the dosing regimens. The contraindications for TXA use were patients with a documented history of thromboembolic events; severe renal impairment or liver insufficiency; allergy to TXA and discontinued IV, or otherwise did not receive TXA.

On arrival to the operating room, an arterial blood sample for blood gas analysis was obtained when patients underwent the radial artery catheterisation. Another arterial blood gas analysis was obtained after prosthesis installation; when patients were admitted to the post-anesthesia care unit following surgery and anesthesia, arterial blood gas analysis was also performed before removing the arterial catheter.

At the very least, 3 arterial blood samples were routinely collected for blood gas analysis. Additional repeated arterial blood gas analyses were drawn based on clinical judgment and values obtained from invasive hemodynamic monitoring. Although arterial blood samples were analyzed immediately on site primarily to determine the amounts of arterial gases, the anesthesiologist also used it to guide specific treatment decisions to correct electrolyte disturbances.

There were no major changes in our perioperative care pathway during the study period.

2.2.4. Outcomes. The main study outcomes are the incidence of acute blood anemia (defined as hemoglobin (Hb) level of <70 g/L or symptomatic anemia) requiring transfusion, and incidence of hypoalbuminemia (defined as albumin level of <30 g/L) requiring albumin supplementation. The secondary outcomes are the rates of acute kidney injury (defined as an increase in baseline serum creatinine [Cre] ≥26.5 μmol/L [≥0.3 mg/dL]), incidence of abnormal serum sodium (Na) level, incidence of abnormal serum potassium (K) level, and incidence of abnormal serum calcium (Ca) level.

2.3. Data collection

2.3.1. Patient characteristics. The following patient data will be collected from the electronic medical record system: age, sex, body weight, height, BMI, surgical indication, preoperative comorbidities (i.e., anemia, hypoalbuminemia, and diabetes), preoperative laboratory values, American Society of Anesthesiologist physical status classification, operation time, intraoperative blood loss, TXA use, thigh tourniquet use, postoperative laboratory values, postoperative length of stay, and any medical intervention directly related to abnormal laboratory values.

2.3.2. Laboratory tests. The laboratory test results collected and analyzed on the first postoperative day are as follows: Hb, hematocrit (Hct), platelet count (PLT), albumin (Alb), Cre, Na, K, and Ca. In our institution, normal laboratory values are defined as follows: Hb (130–175 g/L in males and 115–150 g/L in females), Hct (40%–50% in males and 35%–45% in females), and PLT (85–303*10^9/L in males and 101–320*10^9/L in females) (Table 1); Alb (40–55 g/L), Cre (57–97 μmol/L in males and 41–81 μmol/L in females), Na (137–147 mmol/L), K (3.5–5.3 mmol/L), and Ca (2.11–2.52 mmol/L) (Table 2). An abnormal preoperative or postoperative laboratory test results will be identified if recorded outside of the corresponding reference interval.

Specifically, any medical intervention directly related to abnormal laboratory values are defined as medical or surgical treatments in direct response to postoperative abnormal laboratory test results (eg, anemia prompting blood transfusion, hypoalbuminemia requiring albumin therapy, electrolyte supplementation, hospitalist consultation, addition or withdrawal of medication). In our department, a restrictive red blood cell transfusion protocol mandates a Hb level less than 70 g/L or symptomatic anemia with a Hb level greater than 70 g/L; and the threshold for albumin supplementation is an albumin concentration below 30 g/L.[58] Technically, sodium or potassium supplementation should be given once hyponatremia or hypokalemia is detected on routine postoperative laboratory tests. Hypocalcemia is commonly observed, and calcium supplementation should be administered when the serum Ca level <2.0 mmol/L or patients are symptomatic.

Table 2

Reference ranges for comprehensive metabolic panel and theoretically threshold values for clinical intervention.

	Reference range		
Component	Male	Female	Theoretically threshold values for clinical intervention
Albumin, g/L	40–55	40–55	Albumin level <30 g/L
Creatinine, μmol/L	57–97	41–81	Increase in baseline creatinine ≥26.5 μmol/L
Sodium, mmol/L	137–147	137–147	Sodium level <137 mmol/L
Potassium, mmol/L	3.5–5.3	3.5–5.3	Potassium level <3.5 mmol/L
Calcium, mmol/L	2.11–2.52	2.11–2.52	Calcium level <2.0 mmol/L or if patients are symptomatic

2.3.3. Statistical analysis. Statistical analysis will be performed with the software SPSS for Windows version 21.0 (IBM Corporation, Armonk, NY). Frequencies or percentages will be calculated for qualitative data, and means ± standard deviations or medians (interquartile ranges) will be calculated for quantitative data. The independent *t* test or Wilcoxon rank-sum test will be used to examine differences in continuous variables between groups; while the chi-square test or Fisher exact test will be used to compare differences in categorical variables. Multi-variable regression models will be applied to identify the risk factors in patients with abnormal laboratory test results who required postoperative medical intervention. All baseline differences between the groups will be entered into the logistic regression models. The results are presented using the odds ratio and 95% confidence interval. Statistically significant differences are defined as those with a P value <.05.

2.3.4. Ethics and dissemination. The study will be performed in compliance with the Declaration of Helsinki. Confidentiality of patients' personal information will be protected. Each included patient will be given a study identification number, and data will be collected anonymously, ensuring that participants will not be identified through any data, transcripts, or publications. Researchers interested in testing hypotheses with the data are encouraged to contact the corresponding author. This study forms part of the author's graduation thesis, and results from the study will be disseminated in MD medical theses, and will be assessed by the Chongqing Medical University. The findings of this study will be disseminated at national and international conferences, and will also be published in peer-reviewed, scientific journals.

3. Discussion

The past few decades have witnessed significant improvement in both surgical technique and perioperative care pathways. The concepts of MIS and ERAS in TJA have been quite popular in recent years, and widespread use of TXA has brought revolutionary changes in the field of TJA. There is a growing body of knowledge that postoperative laboratory tests in primary unilateral TJA are only necessary in patients with identified risk factors, and routine postoperative laboratory tests should be discouraged for most patients in modern clinical practice, which impelled us to redefine the necessity of postoperative laboratory tests and re-evaluate the clinical utility of routine practice in the Chinese population.[47–53,59–60] To the best of our knowledge, this study will be the first study in to evaluate the necessity of postoperative laboratory tests for Chinese patients after primary unilateral THA and TKA.

This study is limited by the inherent problems of retrospective studies, in particular, missing, inaccurate or incomplete data. All the patient characteristics, laboratory test results, medical interventions directly related to abnormal laboratory values, and clinical symptoms in patients are inherently reliant upon the documentation in the electronic medical record system. Another major limitation of this study is the variable practice guidelines followed among the surgeons in response to abnormal laboratory values. Thresholds to direct medical intervention varied among surgeons, which might limit the generalizability of the study findings.

In conclusion, this study will provide additional evidence for surgeons regarding the necessity of routine postoperative laboratory tests after primary THA and TKA. The findings of this study will be valuable for improving modern clinical practice, and improving the ERAS program as well.

Author contributions

Conceived and designed the study: Xiang-Dong Wu, and Wei Huang.
Drafted the study protocol: Xiang-Dong Wu, Peng-Cheng Xiao, Zheng-Lin Zhu, Jia-Cheng Liu, Yu-Jian Li, Wei Huang.
Final approval of the version to be published: Xiang-Dong Wu, Peng-Cheng Xiao, Zheng-Lin Zhu, Jia-Cheng Liu, Yu-Jian Li, Wei Huang.
Responsible for data collection, analysis and interpretation: Xiang-Dong Wu, Peng-Cheng Xiao, Zheng-Lin Zhu, Jia-Cheng Liu, Yu-Jian Li, Wei Huang.
Responsible for managing for the project and conducting formal analysis: Xiang-Dong Wu, Peng-Cheng Xiao, Zheng-Lin Zhu, Jia-Cheng Liu, Yu-Jian Li, Wei Huang.
Responsible for study implementation: Xiang-Dong Wu, Peng-Cheng Xiao, Zheng-Lin Zhu, Jia-Cheng Liu, Yu-Jian Li, Wei Huang.
Reviewed and revised the study protocol: Xiang-Dong Wu, Peng-Cheng Xiao, Zheng-Lin Zhu, Jia-Cheng Liu, Yu-Jian Li, Wei Huang.
Conceptualization: Xiang-Dong Wu, Wei Huang.
Data curation: Xiang-Dong Wu, Peng-Cheng Xiao, Zheng-Lin Zhu, Jia-Cheng Liu, Yu-Jian Li, Wei Huang.
Formal analysis: Xiang-Dong Wu, Peng-Cheng Xiao, Zheng-Lin Zhu, Jia-Cheng Liu, Yu-Jian Li, Wei Huang.
Funding acquisition: Wei Huang.
Investigation: Xiang-Dong Wu, Yu-Jian Li.
Methodology: Xiang-Dong Wu, Peng-Cheng Xiao, Zheng-Lin Zhu, Jia-Cheng Liu, Yu-Jian Li, Wei Huang.
Project administration: Xiang-Dong Wu.
Resources: Xiang-Dong Wu, Zheng-Lin Zhu, Wei Huang.
Software: Xiang-Dong Wu, Peng-Cheng Xiao, Zheng-Lin Zhu, Jia-Cheng Liu, Yu-Jian Li, Wei Huang.
Supervision: Xiang-Dong Wu, Wei Huang.
Validation: Xiang-Dong Wu, Peng-Cheng Xiao, Zheng-Lin Zhu, Jia-Cheng Liu, Yu-Jian Li, Wei Huang.
Writing – original draft: Xiang-Dong Wu, Peng-Cheng Xiao, Zheng-Lin Zhu, Jia-Cheng Liu, Yu-Jian Li, Wei Huang.
Writing – review and editing: Xiang-Dong Wu, Wei Huang.

References

[1] Harris WH, Sledge CB. Total hip and total knee replacement. N Engl J Med 1990;323:725–31.
[2] Learmonth ID, Young C, Rorabeck C. The operation of the century: total hip replacement. Lancet 2007;370:1508–19.
[3] English T. The trochanteric approach to the hip for prosthetic replacement. J Bone Joint Surg Am Vol 1975;57:1128–33.
[4] Wright JM, Crockett HC, Delgado S, et al. Mini-incision for total hip arthroplasty: a prospective, controlled investigation with 5-year follow-up evaluation. J Arthroplasty 2004;19:538–45.
[5] Wenz JF, Gurkan I, Jibodh SR. Mini-incision total hip arthroplasty: a comparative assessment of perioperative outcomes. Orthopedics 2002;25:1031–43.
[6] DiGioia III AM, Plakseychuk AY, Levison TJ, et al. Mini-incision technique for total hip arthroplasty with navigation. J Arthroplasty 2003;18:123–8.
[7] Bierbaum BE, Callaghan JJ, Galante JO, et al. An analysis of blood management in patients having a total hip or knee arthroplasty. JBJS 1999;81:2–10.
[8] Helm A, Karski M, Parsons S, et al. A strategy for reducing blood-transfusion requirements in elective orthopaedic surgery: audit of an algorithm for arthroplasty of the lower limb. J Bone Joint Surg Br Vol 2003;85:484–9.
[9] Lemaire R. Strategies for blood management in orthopaedic and trauma surgery. J Bone Joint Surg Br Vol 2008;90:1128–36.

[10] Surgenor D, Wallace E, Churchill W, et al. Red cell transfusions in total knee and total hip replacement surgery. Transfusion 1991;31:531–7.
[11] Eriksson BI, Borris LC, Friedman RJ, et al. Rivaroxaban versus enoxaparin for thromboprophylaxis after hip arthroplasty. N Engl J Med 2008;358:2765–75.
[12] Kakkar AK, Brenner B, Dahl OE, et al. Extended duration rivaroxaban versus short-term enoxaparin for the prevention of venous thromboembolism after total hip arthroplasty: a double-blind, randomised controlled trial. Lancet 2008;372:31–9.
[13] Ralley FE, Berta D, Binns V, et al. One intraoperative dose of tranexamic acid for patients having primary hip or knee arthroplasty. Clin Orthop Relat Res 2010;468:1905–11.
[14] Friedman R, Homering M, Holberg G, et al. Allogeneic blood transfusions and postoperative infections after total hip or knee arthroplasty. JBJS 2014;96:272–8.
[15] SooHoo NF, Lieberman JR, Ko CY, et al. Factors predicting complication rates following total knee replacement. JBJS 2006;88: 480–5.
[16] Parvizi J, Mui A, Hozack WJ, et al. Total joint arthroplasty: when do fatal or near-fatal complications occur? J Bone Joint Surg Am 2007;89:27–32.
[17] Forrest G, Fuchs M, Gutierrez A, et al. Factors affecting length of stay and need for rehabilitation after hip and knee arthroplasty. J Arthroplasty 1998;13:186–90.
[18] Munin MC, Rudy TE, Glynn NW, et al. Early inpatient rehabilitation after elective hip and knee arthroplasty. JAMA 1998;279:847–52.
[19] Repicci JA, Eberle R. Minimally invasive surgical technique for unicondylar knee arthroplasty. J South Orthop Assoc 1999;8:20–7.
[20] Hong CM. Minimally invasive joint replacement surgery: where are we now? J Orthop Case Rep 2017;7:3–4.
[21] Bonutti PM, Mont MA, McMahon M, et al. Minimally invasive total knee arthroplasty. JBJS 2004;86((suppl_2)):26–32.
[22] Wu X-D, Chen Y, Wang Z-Y, et al. Comparison of periprosthetic bone remodeling after implantation of anatomic and tapered cementless femoral stems in total hip arthroplasty: a prospective cohort study protocol. Medicine 2018;97:e12560.
[23] Wang T, Shao L, Xu W, et al. Surgical injury and repair of hip external rotators in THA via posterior approach: a three-dimensional MRI-evident quantitative prospective study. BMC Musculoskelet Disord 2019;20:22.
[24] Eum DS, Lee HK, Hwang SY, et al. Blood loss after navigation-assisted minimally invasive total knee arthroplasty. Orthopedics 2006;29:S152–4.
[25] Chang CW, Wu PT, Yang CY. Blood loss after minimally invasive total knee arthroplasty: effects of imageless navigation. Kaohsiung J Med Sci 2010;26:237–43.
[26] Wu X-D, Xiang B-Y, Schotanus MG, et al. CT-versus MRI-based patient-specific instrumentation for total knee arthroplasty: a systematic review and meta-analysis. Surgeon 2017;15:336–48.
[27] Jones CW, Jerabek SA. Current role of computer navigation in total knee arthroplasty. J Arthroplasty 2018;33:1989–93.
[28] Xu C-P, Li X, Song J-Q, et al. Mini-incision versus standard incision total hip arthroplasty regarding surgical outcomes: a systematic review and meta-analysis of randomized controlled trials. PloS One 2013;8:e80021.
[29] Migliorini F, Biagini M, Rath B, et al. Total hip arthroplasty: minimally invasive surgery or not? Meta-analysis of clinical trials. Int Orthop 2018;[Epub ahead of print]. https://www.ncbi.nlm.nih.gov/pubmed/30171273.
[30] King J, Stamper DL, Schaad DC, et al. Minimally invasive total knee arthroplasty compared with traditional total knee arthroplasty. Assessment of the learning curve and the postoperative recuperative period. J Bone Joint Surg Am 2007;89:1497–503.
[31] Zawadsky MW, Paulus MC, Murray PJ, et al. Early outcome comparison between the direct anterior approach and the mini-incision posterior approach for primary total hip arthroplasty: 150 consecutive cases. J Arthroplasty 2014;29:1256–60.
[32] Sershon RA, Tetreault MW, Della Valle CJ. A prospective randomized trial of mini-incision posterior and 2-incision total hip arthroplasty: minimum 5-year follow-up. J Arthroplasty 2017;32:2462–5.
[33] Kehlet H. Multimodal approach to control postoperative pathophysiology and rehabilitation. Br J Anaesth 1997;78:606–17.
[34] Kehlet H, Wilmore DW. Multimodal strategies to improve surgical outcome. Am J Surg 2002;183:630–41.
[35] Deng Q-F, Gu H-Y, Peng W-Y, et al. Impact of enhanced recovery after surgery on postoperative recovery after joint arthroplasty: results from a systematic review and meta-analysis. Postgrad Med J 2018;94:678–93.
[36] Kehlet H. Fast-track hip and knee arthroplasty. Lancet 2013;381:1600–2.
[37] Aasvang E, Luna I, Kehlet H. Challenges in postdischarge function and recovery: the case of fast-track hip and knee arthroplasty. Br J Anaesth 2015;115:861–6.
[38] Soffin E, YaDeau J. Enhanced recovery after surgery for primary hip and knee arthroplasty: a review of the evidence. Br J Anaesth 2016;117 ((suppl_3)):iii62–72.
[39] Barbieri A, Vanhaecht K, Van Herck P, et al. Effects of clinical pathways in the joint replacement: a meta-analysis. BMC Med 2009;7:32.
[40] Kumar L, Kumar AH, Grant SA, et al. Updates in enhanced recovery pathways for total knee arthroplasty. Anesthesiol Clin 2018;36:375–86.
[41] Fillingham YA, Ramkumar DB, Jevsevar DS, et al. Tranexamic acid use in total joint arthroplasty: the clinical practice guidelines endorsed by the American Association of Hip and Knee Surgeons, American Society of Regional Anesthesia and Pain Medicine, American Academy of Orthopaedic Surgeons, Hip Society, and Knee Society. Arthroplasty 2018;33:3065–9.
[42] Tan J, Chen H, Liu Q, et al. A meta-analysis of the effectiveness and safety of using tranexamic acid in primary unilateral total knee arthroplasty. J Surg Res 2013;184:880–7.
[43] Wu X-D, Hu K-J, Sun Y-Y, et al. Letter to the editor on "the safety of tranexamic acid in total joint arthroplasty: a direct meta-analysis". J Arthroplasty 2018;33:3365–8.
[44] Fillingham YA, Ramkumar DB, Jevsevar DS, et al. The safety of tranexamic acid in total joint arthroplasty: a direct meta-analysis. J Arthroplasty 2018;33:3070–82.
[45] Fillingham YA, Ramkumar DB, Jevsevar DS, et al. The efficacy of tranexamic acid in total hip arthroplasty: a network meta-analysis. J Arthroplasty 2018;33:3083–9.
[46] Fillingham YA, Ramkumar DB, Jevsevar DS, et al. The efficacy of tranexamic acid in total knee arthroplasty: a network meta-analysis. J Arthroplasty 2018;33:3090–8.
[47] Shaner JL, Karim AR, Casper DS, et al. Routine postoperative laboratory tests are unnecessary after partial knee arthroplasty. J Arthroplasty 2016;31:2764–7.
[48] Kildow BJ, Karas V, Howell E, et al. The utility of basic metabolic panel tests after total joint arthroplasty. J Arthroplasty 2018;33:2752–8.
[49] Halawi MJ, Lyall V, Cote MP. Re-evaluating the utility of routine postoperative laboratory tests after primary total knee arthroplasty. J Clin Orthop Trauma 2019;[Epub ahead of print]. https://www.sciencedirect.com/science/article/pii/S0976566218306817.
[50] Halawi MJ, Plourde JM, Cote MP. Routine postoperative laboratory tests are not necessary after primary total hip arthroplasty. J Arthroplasty 2019;34:538–41.
[51] Howell EP, Kildow BJ, Karas V, et al. Clinical impact of routine complete blood counts following total knee arthroplasty. J Arthroplasty 2019;[Epub ahead of print]. https://www.ncbi.nlm.nih.gov/pubmed/30948289.
[52] Greco N, Manocchio A, Lombardi A, et al. Should postoperative haemoglobin and potassium levels be checked routinely following blood-conserving primary total joint arthroplasty? Bone Joint J 2019;101 ((1_Supple_A)):25–31.
[53] Wu XD, Jiang F, Xiang BY, et al. Letter to the editor on "routine postoperative laboratory tests are not necessary after primary total hip arthroplasty". J Arthroplasty 2019;[Epub ahead of print]. https://www.ncbi.nlm.nih.gov/pubmed/30745216.
[54] Von Elm E, Altman DG, Egger M, et al. STROBE initiative. The strengthening the reporting of observational studies in epidemiology (STROBE) statement: guidelines for reporting observational studies. J Clin Epidemiol 2008;11:344–9.
[55] Carling MS, Jeppsson A, Eriksson BI, et al. Transfusions and blood loss in total hip and knee arthroplasty: a prospective observational study. J Orthop Surg Res 2015;10:48.
[56] Bohl DD, Ondeck NT, Darrith B, et al. Impact of operative time on adverse events following primary total joint arthroplasty. J Arthroplasty 2018;33:2256–62.
[57] Wu X-D, Hu K-J, Tian M, et al. Letter to the editor on "impact of operative time on adverse events following primary total joint arthroplasty". J Arthroplasty 2018;33:2701–2.
[58] Gu W-J, Gu X-P, Wu X-D, et al. Restrictive versus liberal strategy for red blood-cell transfusion: a systematic review and meta-analysis in orthopaedic patients. JBJS 2018;100:686–95.
[59] Lin JM, Cao ZY, Peng AF, et al. Are routine postoperative laboratory tests really necessary after lumbar spinal surgery? World Neurosurg 2019; [Epub ahead of print]. https://www.ncbi.nlm.nih.gov/pubmed/30677571.
[60] Faulkner A, Reidy M, McGowan J. Should we abandon routine blood tests? BMJ 2017;357:j2091.
