# Supplementary material for: The necessity of routine postoperative laboratory tests after total hip arthroplasty for hip fracture in a semi-urgent clinical setting
Source: J Orthop Traumatol. 2020 Nov 10;21:19. doi: 10.1186/s10195-020-00559-3 (PMC7655881; doi:10.1186/s10195-020-00559-3)
Supplement: Supplementary file 2 — Additional file 2: Material S2. The necessity of routine postoperative laboratory tests for enhanced recovery after surgery for primary hip and knee arthroplasty. [file 10195_2020_559_MOESM2_ESM.doc]

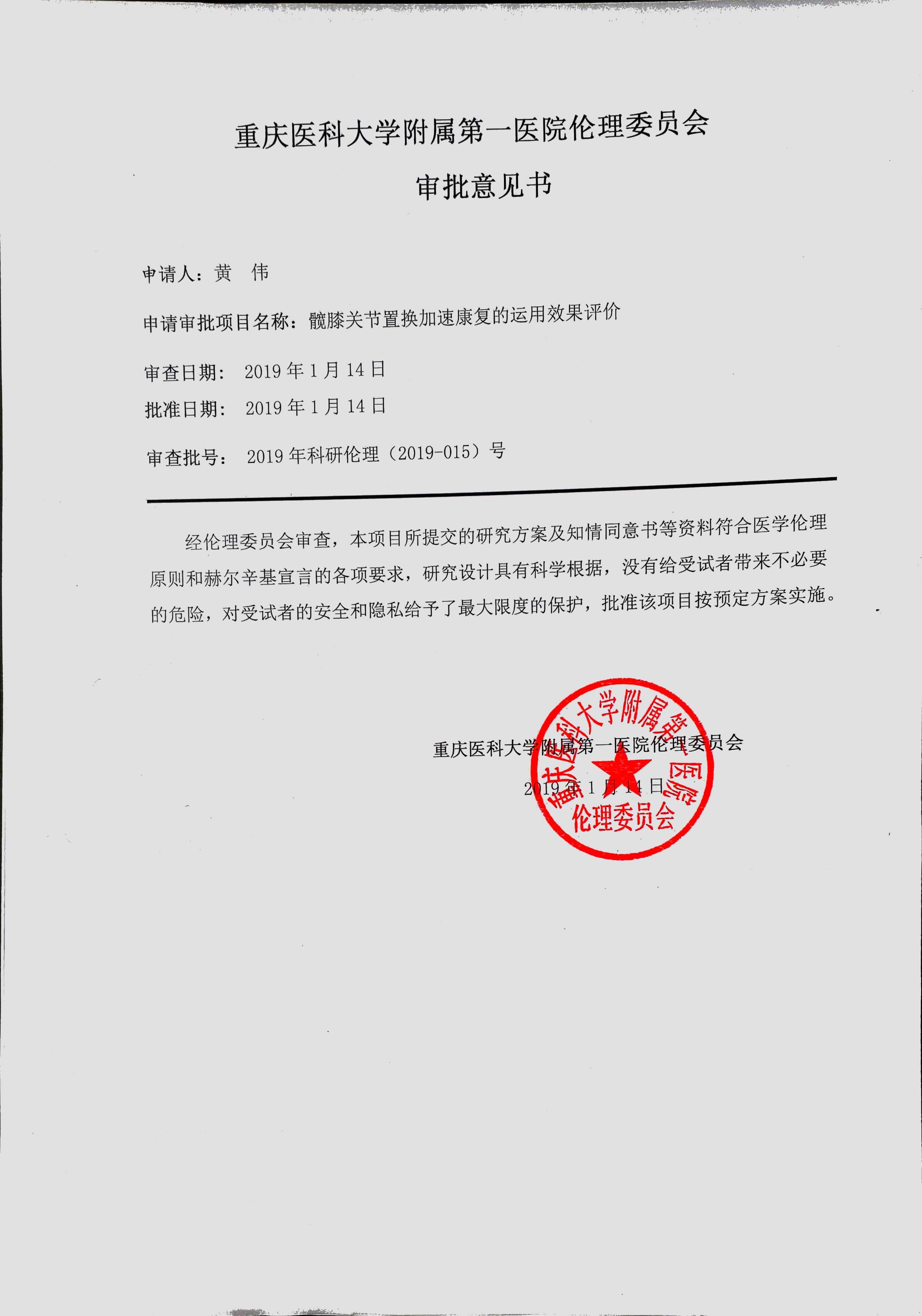


IRB Approval

**Applicant:** Wei Huang

**Title of Project:** Implementation of an Enhanced Recovery After Surgery Program in Total Hip/Knee Arthroplasty: Effectiveness Evaluation

**Approval Date:** January 14^th^ 2019

**Approval Number:** 2019-015

**Approval from:** The Ethics Committee of The First Affiliated Hospital of Chongqing Medical University
