# Supplementary material for: The necessity of routine postoperative laboratory tests after total hip arthroplasty for hip fracture in a semi-urgent clinical setting
Source: J Orthop Traumatol. 2020 Nov 10;21:19. doi: 10.1186/s10195-020-00559-3 (PMC7655881; doi:10.1186/s10195-020-00559-3)
Supplement: Supplementary file 3 — Additional file 3: Material S3. IRB approval. [file 10195_2020_559_MOESM3_ESM.doc]

**Supplementary Material 1. Reference ranges for complete blood count and comprehensive metabolic panel and corresponding threshold values for clinical intervention.**

| **Blood test** | **Component** | **Reference Range** | | **Threshold values for clinical intervention** |
| --- | --- | --- | --- | --- |
|  |  | **Male** | **Female** |  |
| Complete blood count (CBC) | Hemoglobin (g/L) | 130~175 | 115~150 | Hemoglobin level of <70 g/L or symptomatic anemia with a hemoglobin level > 70 g/L |
|  | Platelet count (10^9^/L) | 85~303 | 101~320 | Platelet count < 30*10^9^/L or poor platelet function |
| Comprehensive metabolic panel (CMP) | Albumin (g/L) | 40~55 | 40~55 | Albumin level <30 g/L |
|  | Creatinine (μmol/L) | 57~97 | 41~81 | Increase in baseline creatinine ≥26.5 μmol/L |
|  | Sodium (mmol/L) | 137~147 | 137~147 | Sodium level <137mmol/L |
|  | Potassium (mmol/L) | 3.5~5.3 | 3.5~5.3 | Potassium level <3.5mmol/L |
|  | Calcium (mmol/L) | 2.11~2.52 | 2.11~2.52 | Calcium level < 2.0 mmol/L or if patients are symptomatic |
